# Lost in digitization – A systematic review about the diagnostic test accuracy of digital pathology solutions

**DOI:** 10.1016/j.jpi.2022.100136

**Published:** 2022-09-06

**Authors:** Olsi Kusta, Charlotte Vestrup Rift, Torsten Risør, Eric Santoni-Rugiu, John Brandt Brodersen

**Affiliations:** a Department of Public Health, University of Copenhagen, Øster Farimagsgade 5 opg. B, Building: 15-0-11, 1014 Copenhagen, Denmark; b Centre for Research in Assessment and Digital Learning (CRADLE), Deakin University, Melbourne, Australia; c Department of Pathology, Rigshospitalet (Copenhagen University Hospital), Blegdamsvej 9, 2100 Copenhagen, Denmark; d Centre for General Practice, Department of Public Health, University of Copenhagen, Øster Farimagsgade 5 opg. Q, Building: 24-1, 1014 Copenhagen, Denmark; e Norwegian Centre for E-health Research, UiT The Arctic University of Norway, Tromsø, Norway; f Department of Pathology, Rigshospitalet (Copenhagen University Hospital), Blegdamsvej 9, 2100 Copenhagen, Denmark; g Department of Clinical Medicine, University of Copenhagen, Blegdamsvej 9, 2100 Copenhagen, Denmark; h Centre for General Practice, Department of Public Health, University of Copenhagen, Øster Farimagsgade 5 opg. Q, Building: 24-1-21, 1014 Copenhagen, Denmark; i Primary Health Care Research Unit, Region Zealand, Øster Farimagsgade 5 opg. Q, Building: 24-1-21, 1014 Copenhagen, Denmark.

**Keywords:** Human pathology, Whole slide imaging (WSI), Validation studies, Diagnostic test accuracy, Diagnostic concordance, Overdiagnosis

## Abstract

**Introduction:**

Digital pathology solutions are increasingly implemented for primary diagnostics in departments of pathology around the world. This has sparked a growing engagement on validation studies to evaluate the diagnostic performance of whole slide imaging (WSI) regarding safety, reliability, and accuracy. The aim of this review was to evaluate the performance of digital pathology for diagnostic purposes compared to light microscopy (LM) in human pathology, based on validation studies designed to assess such technologies.

**Methods:**

In this systematic review based on PRISMA guidelines, we analyzed validation studies of WSI compared with LM. We included studies of diagnostic performance of WSI regarding diagnostic test accuracy (DTA) indicators, degree of overdiagnosis, diagnostic concordance, and observer variability as a secondary outcome. Overdiagnosis is (for example) detecting a pathological condition that will either not progress or progress very slowly. Thus, the patient will never get symptoms from this condition and the pathological condition will never be the cause of death. From a search comprising four databases: PubMed, EMBASE, Cochrane Library, and Web of Science, encompassing the period 2010–2021, we selected and screened 12 peer-reviewed articles that fulfilled our selection criteria. Risk of bias was conducted through QUADAS-2 tool, and data analysis and synthesis were performed in a qualitative format.

**Results:**

We found that diagnostic performance of WSI was not inferior to LM for DTA indicators, concordance, and observer variability. The degree of overdiagnosis was not explicitly reported in any of the studies, while the term itself was used in one study and could be implicitly calculated in another.

**Conclusion:**

WSI had an overall high diagnostic accuracy based on traditional accuracy measurements; however, the degree of overdiagnosis is unknown.

## Highlights

• Diagnostic accuracy of whole slide imaging is not inferior to light microscopy.
• Traditional accuracy measurements should also include overdiagnosis.
• Overdiagnosis might artificially increase the performance of whole slide imaging.
• Heterogeneity of pathology subspecialties might affect diagnostic performance.

## Introduction

In the era of precision medicine, pathology departments face multiple challenges in relation to the complexity of companion diagnostics, and strict deadlines for timely diagnoses within cancer, chronic inflammatory, and degenerative diseases,^1^ yielding an increased workload. Many departments in different countries are using digital pathology for their routine work as one potential solution to the above challenges.^2^ In Denmark, for instance, healthcare policy documents claim that this digital solution could facilitate faster response rates, better collaboration with clinicians, and in the future the opportunity to use artificial intelligence to assist diagnosis.^3^

Digital pathology, based on whole slide imaging (WSI) technologies, encompasses mainly 3 major components: information systems, image management system (IMS), and image analysis tools.^4^ There are several advantages of using WSI for clinical purposes, such as fast consultations (specialists providing second opinions or supervision of residents), remote interpretation of frozen sections in surgical pathology, and telepathology for primary diagnosis.^5^ Other advantages that make digital pathology appealing are biomarker research^6^ and the potential advantages of using artificial intelligence (AI).^7^

Using this technology for in vitro diagnostics (IVD), entails a validation process regarding the reliability, safety, and accuracy of these devices.^8^ The new European regulation for IVD medical devices (2017/746), stipulates that they require a performance evaluation to be approved for clinical use. This evaluation entails 3 main reported steps: scientific validity, analytical performance, and clinical performance.^8^ The latter is based on diagnostic test accuracy (DTA) indicators as also elaborated in the Cochrane collaboration.^9^ The most commonly referred measures of DTA are sensitivity, specificity, predictive values (of negative or positive test results), likelihood ratios, receiver operating characteristics (ROC) curves, and area under the ROC curve (AUC).

The Food and Drug Agency^10^ (FDA) puts forth additional guidelines for the validation process of WSI based on College of American Pathologists (CAP) recommendations,^11^ such as pathologists trained with WSI, a representative number of cases, an adequate time interval between the use of LM and WSI for the same case, diagnostic concordance (i.e., intraobserver variability), and that all the material in the glass slide is present in the digital format. In the evaluation and approval of the Philips IntelliSite Pathology Solution (PIPS), FDA considered the diagnostic concordance (96.5%) of WSI as non-inferior to LM in the clinical performance report.^12^ We have selected the studies for review based on the accuracy measurements as elaborated in both European and US regulations.

However, the use of devices with high resolution potentially introduces a risk of overdiagnosis. Overdiagnosis is detecting a cancer, for instance, that will not progress (or progress very slowly) to harm the patient or be the cause of death.^13^ In relation to high resolution imaging devices, the presence of overdiagnosis will cause the sensitivity and the positive-predictive value to be artificially inflated. If there is a substantial risk of overdiagnosis, the traditional DTA measures would be distorted resulting in biased performance of the diagnostic test.^14^^,^^15^ The main problem is that overdiagnosis cannot be captured in the traditional accuracy measurements based on the Bayesian (2x2) table as misdiagnosis or underdiagnosis, as it fulfills the pathological criteria of abnormality.^16^

Therefore, our research question was: what is the diagnostic performance, including the degree of overdiagnosis, of WSI compared to conventional LM? Thus, the aim of this study was to evaluate the performance through diagnostic test accuracy (DTA) indicators, degree of overdiagnosis, diagnostic concordance, and observer variability as a secondary outcome. This was done through a systematic review of validation studies of WSI versus LM.

## Materials and methods

**Fig. 1:**
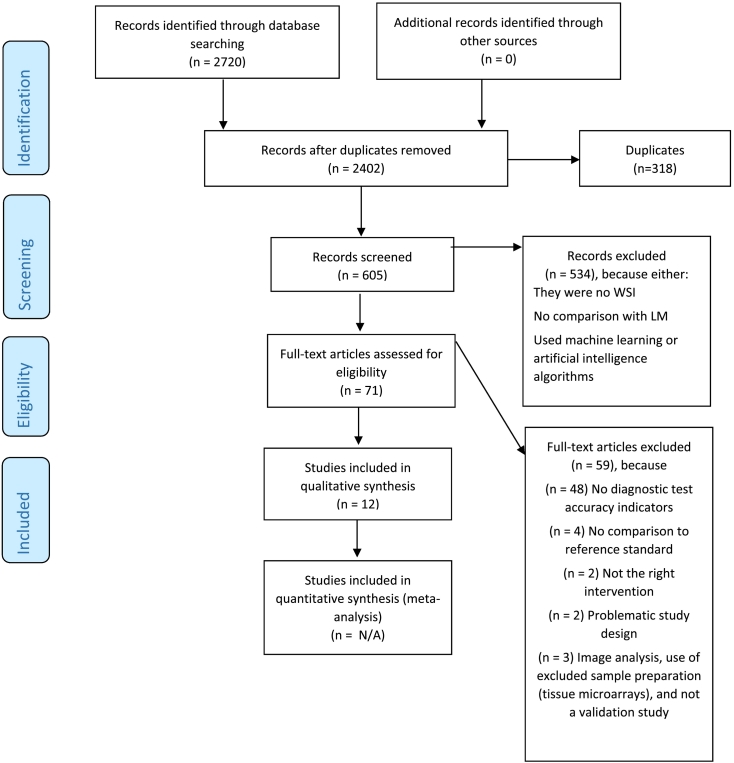
Flowchart based on Preferred Reporting Items for Systematic Reviews and Meta-Analyses (PRISMA^a^) guidelines. ^a^The figure was drafted based on a freely available template at http://prisma-statement.org/documents/PRISMA%202009%20flow%20diagram.pdf.

This systematic review was based on PRISMA-P guidelines,^17^ with the protocol registered in PROSPERO (CRD42021243403). A PRISMA flow diagram was created to present the selection process for this systematic review (Fig. 1). Two authors (CVR and OK), independently from each other, screened the databases, extracted the data, assessed the quality of the studies, analyzed, and provided a synthesis for the results. In cases of disagreements during these steps, JBB was consulted to arbitrate for these cases.

The evaluation of WSI versus LM, was based on 3 main outcomes: DTA indicators,^9^ diagnostic concordance, and degree of overdiagnosis. For the latter, we screened for its 2 main causes: overdetection and overdefinition. The first is defined as finding pathological abnormalities that will never progress to do any harm or progress very slowly, thus not being the cause of death.^16^ Overdefinition, the other subtype, can either be lowering the threshold for a risk factor without evidence of any benefical effects or expanding the disease definition including, e.g., milder symptoms.^16^ The additional outcome included here was observer variability.

Our focus was only on human pathology, including all the tissue specimen preparations such as biopsies, resected specimens, frozen sections, and cytology samples; and all the stains used for diagnostic purposes, such as hematoxylin and eosin (HE), immunohistochemical stains (IHC), and special stains. Only WSI systems were considered and no additional system tools, i.e., image analysis algorithms.^4^ We included only peer-reviewed articles regarding clinical evaluation or validation studies and no gray literature.

We searched 4 databases during May, and August–October 2021: PubMed, EMBASE, Cochrane Library, and Web of Science – including articles published during the period 2010–2021. The main simplified search string was: Digital Pathology (whole slide imaging OR digital microscope OR virtual microscope) OR Digital Slides (digitized slides OR virtual slides) AND Diagnostic Accuracy (DTA OR diagnostic performance OR accuracy) AND NOT Image Processing, Computer Assisted [Mesh terms] (machine learning OR artificial intelligence OR algorithms).

The quality of the selected studies was assessed through the modified Quality Assessment of Diagnostic Accuracy Studies (QUADAS-2) tool.^18^ The assessment of bias in the studies was based on 4 domains: patient selection, index test, reference standard, flow of patients in the study, and timing of the intervention(s).^19^

Primary and secondary outcomes are reported in a tabular form, while the other data extracted as supplementary material. We did not conduct a meta-analysis because of the studies heterogeneity.

## Results

### Study characteristics and quality assessment

We identified 2402 unique records in our literature search of which 71 articles were included for full text reading and possible elegibility for the study (Fig. 1). Among the 71 articles, 12 fulfilled the main selection criteria for our study that is reporting at least 2 of the primary outcomes (i.e., DTA indicators, diagnostic concordance, and overdiagnosis). From the 12 studies in our review, 4 did not specify the kind of study^20^, ^21^, ^22^, ^23^; 3 were retrospective studies,^24^, ^25^, ^26^ 2 comparative studies,^27^^,^^28^ and the remaining 3 randomized,^29^ evaluation,^30^ and validation study,^31^ respectively. The characteristics of the studies are presented in the Supplementary Tables 1 and 2.

Of emphasis concerning digitization of slides is that only 2 studies reported minor technical discrepancies. One study elaborated on a technical issue where 11 of 124 slides needed a rescan and 4 were excluded due to failed digitization^31^; while another stated that 6 slides had loss of diagnostic material on the fine needle biopsy.^21^ The most used WSI scanner as reported in 4 studies, was Aperio ScanScope XT (Aperio Technologies, Vista, Calif., USA),^22^^,^^24^^,^^26^^,^^28^ followed by iScan Coreo (Ventana, Tucson, Ariz., USA) used in 3 studies.^20^^,^^23^^,^^29^ In the remaining studies, there were diverse scanners used such as Mirax scanner (Carl Zeiss MicroImaging, Jena, Germany),^25^^,^^30^ NanoZoomer S260 (Hamamatsu photonics, Japan),^27^ Navigo (Visia Imaging, Arezzo, Italy),^31^ and digital camera with NetCam software (Olympus America, Center Valley, PA).^21^

**Fig. 2:**
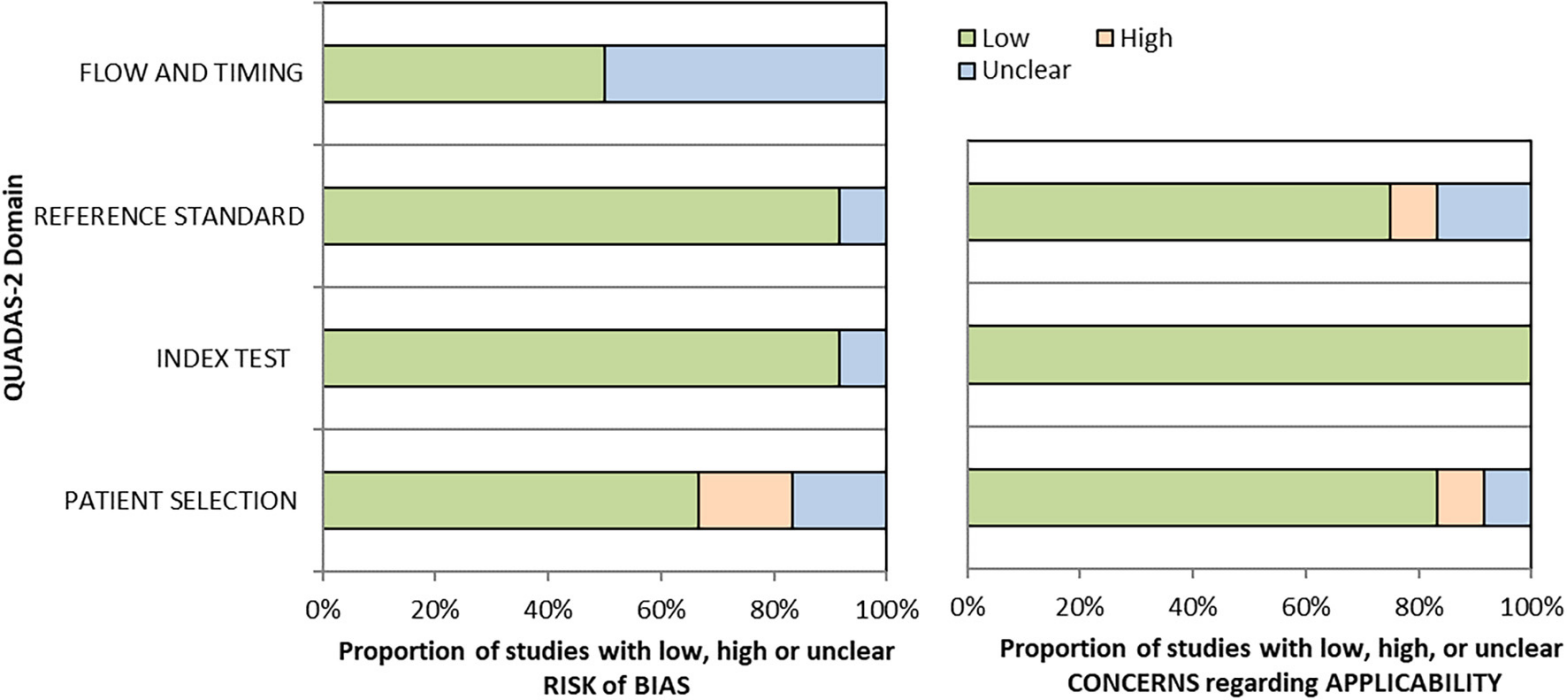
The proportion of the Risk of Bias and Applicability Concerns (QUADAS 2)^a^. ^a^The drafted figure is a template freely available at https://view.officeapps.live.com/op/view.aspx?src=http%3A%2F%2Fwww.bristol.ac.uk%2Fmedia-library%2Fsites%2Fquadas%2Fmigrated%2Fdocuments%2Fgraphs.xlsx&wdOrigin=BROWSELINK.

Regarding the quality assessment of the selected studies, overall there was a low risk of bias and applicability concerns (for more details see Table 1, Table 2, and Fig. 2).

**Table 1:** Judgement for Risk of Bias summarized for domains (QUADAS 2)^a^.

Authors	Patient selection	Index test	Reference standard	Flow and timing
Ammendola et al. ^27^	?	?		
Brunyé et al.^20^				
Cima et al. ^31^				
Elmore et al. ^29^				
Larghi et al. ^24^				
Nielsen et al. ^30^			?	
Perez et al. ^21^				?
Ribback et al. ^25^				?
Tawfik et al. ^28^				?
Tawfik et al. ^26^				?
Tissier et al. ^22^	?			?
Zoroquiain et al. ^23^				?

a Table adapted from the freely available template at https://view.officeapps.live.com/op/view.aspx?src=http%3A%2F%2Fwww.bristol.ac.uk%2Fmedia-library%2Fsites%2Fquadas%2Fmigrated%2Fdocuments%2Ftable.docx&wdOrigin=BROWSELINK.

**Table 2:** Applicability concerns for the respective domains (QUADAS 2)^a^.

Authors	Patient selection	Index test	Reference standard
Ammendola et al. ^27^			
Brunyé et al. ^20^			
Cima et al. ^31^			?^b^
Elmore et al. ^29^			
Larghi et al. ^24^			
Nielsen et al. ^30^			
Perez et al. ^21^			
Ribback et al. ^25^			
Tawfik et al. ^28^			^c^
Tawfik et al. ^26^			
Tissier et al. ^22^	?		?
Zoroquiain et al. ^23^			

a Table adapted from the freely available templates at https://view.officeapps.live.com/op/view.aspx?src=http%3A%2F%2Fwww.bristol.ac.uk%2Fmedia-library%2Fsites%2Fquadas%2Fmigrated%2Fdocuments%2Ftable.docx&wdOrigin=BROWSELINK.

b Because final FS-FFPE diagnosis based on frozen sections (FS) or formalin-fixed and paraffin embedded (FFPE) biopsies may differ from the original assessment even during routine use of LM with frozen section.

c This refers to the comparison of accuracy of WSI with LM to identify microorganisms and not human cells.

### Primary and additional outcomes

The primary outcomes that we extracted concerning diagnostic performance of WSI were DTA indicators, diagnostic concordance, and degree of overdiagnosis. As emphasized earlier, the main criteria for selecting the studies was the combination of at least 2 of these outcomes. The additional outcome that is the observer variability, was extracted as an important accuracy measure for validating WSI as elaborated by CAP guidelines.^11^ Four studies reported on the diagnostic performance of both LM and WSI.^24^^,^^27^^,^^29^^,^^30^ Below, we describe briefly these outcomes.

#### Diagnostic test accuracy indicators

The main DTA indicators reported for WSI in 10 studies were sensitivity, specificity, positive-predictive values, and negative-predictive values while in 1 study AUC was reported as a probability.^27^ One study did not specify any DTA indicators, but only diagnostic concordance.^20^ From the 12 selected studies, 5 were based on histology preparations,^22^^,^^23^^,^^27^^,^^29^^,^^30^ 3 used cytology preparations,^21^^,^^26^^,^^28^ 1 study both histology and cytology samples,^24^ while 2 of them frozen sections.^25^^,^^31^ The studies selected encompassed several pathology subspecialties, with 2 of them reporting on multiple^25^^,^^31^ and 1 not specifying the subspecialty.^21^

All the results regarding the primary outcomes of accuracy measurements are shown in Table 3. At least 7 studies reported a very good performance of WSI based on DTA indicators.^21^, ^22^, ^23^, ^24^, ^25^, ^26^^,^^30^^,^^31^ In these studies, sensitivity ranged from 86% to 100%, specificity 75% to 100%, positive-predictive values 92% to 99%, and negative-predictive values from 75% to 100%. Cima et al., examining frozen sections for intraoperative cancer staging and transplant organs, had a drop in specificity and negative-predictive values (both 75%), due to 4 discordant cases (compared to LM) in examining kidney and liver donors transplant organs.^31^

**Table 3:** Primary outcomes of diagnostic test accuracy (DTA) indicators and diagnostic concordance.

Source	Subspecialty	Diagnostic purpose	Primary outcomes								

Ammendola et al.^27^	Surgical Neuropathology	Grading of meningioma	*Area Under the Curve (AUC)*^a^								

				Observer 1		Observer 2		Observer 3		Observer 4	
			**Histopathological features**^b^	**LM**	**WSI**	**LM**	**WSI**	**LM**	**WSI**	**LM**	**WSI**

			Brain invasion	0.50	0.50	0.51	0.51	0.53	0.55	0.50	0.55
			High mitotic index	0.64	0.72	0.60	0.61	0.58	0.65	0.56	0.68
			Hypercellularity	0.54	0.52	0.58	0.58	0.50	0.50	0.54	0.50
			Sheeting	0.57	0.52	0.59	0.59	0.55	0.59	0.50	0.62
			Macronucleoli	0.53	0.51	0.55	0.53	0.51	0.53	0.53	0.53
Small cells	0.55	0.51	0.63	0.61	0.54	0.53	0.52	0.54
Spontaneous necrosis	0.51	0.52	0.61	0.61	0.51	0.51	0.56	0.54

Brunyé et al.^20^	Breast pathology	Classification of breast neoplasms	*Diagnostic concordance (95% CI)*								

			**Consensus diagnosis**	**Mean concordance**		**Above**^c^			**Below**^d^		

			Benign	71% (61–82%)		29% (20–40%)			-		
			Atypia	37% (29–45%)		21% (15–28%)			43% (35–50%)		
Ductal Carcinoma in Situ (DCIS)	52% (43–61%)		17% (12–23%)			31% (25–39%)		
Invasive breast cancer	94% (88–99%)		–			6% (2–14%)		

Cima et al.^31^	Multiple subspecialties and organs	Cancer staging (surgical margins, tumor biology, lymph node status) and organ quality for transplantation	**Primary outcomes**		**Cancer (WSI)**			**Transplant (WSI)**			

			*Sensitivity*		100%			96%			
			*Specificity*		96%			75%			
			*Positive-predictive values*		95%			96%			
			*Negative-predictive values*		100%			75%			
*Diagnostic concordance*		97% (к=0.96, CI: 0.941–0.985)			86% (к=0.91, CI: 0.877–0.958)			

Elmore et al.^29^	Breast pathology	Diagnosis of breast cancer	*Predictive values*								

			**Pathologist interpretation**^e^		**LM (95% CI)**			**WSI (95% CI)**			
			Benign without atypia		97.1% (96.7–97.4%)			95.7% (95.0–96.4%)			
			Atypia		37.8% (33.6–42.7%)			27.8% (23.9–32.5%)			
			Ductal Carcinoma in situ (DCIS)		69.6% (64.4–75.3%)			57.1% (50.6–64.8%)			
Invasive breast cancer		97.7% (96.5–98.7%)			97.2% (95.6–98.6%)			

Larghi et al.^24^	Pancreatic pathology	Diagnostic classification according to the Papanicolau Society of Cytopathology system for reporting pancreatobiliary cytology	**Primary outcomes**		**LM (95% CI)**			**WSI (95% CI)**			

			*Sensitivity*		92%			93%			
			*Specificity*		96%			88%			
			*Positive-predictive values*		99%			99%			
			*Negative-predictive values*		51%			52%			
*Diagnostic concordance*		92%			92%			

Nielsen et al.^30^	Dermatopathology	Diagnosing neoplasms of the skin: benign, premalignant, and malignant	**Primary outcomes**		**LM**			**WSI**			
			*Sensitivity*		92% (85–96%)			86% (78–91%)			
			*Specificity*		99.5% (97–99.5%)			99% (97–99.5%)			
			*Positive-predictive values*		93% (86–96.5%)			92% (84.5–95.5%)			
			*Negative-predictive values*		98% (97–99%)			97% (96–98%)			
			*Diagnostic concordance*^f^		72.4%			69.6%			
Perez et al.^21^	Not specified	Diagnosing neoplasms: benign, suspicious, and malignant	**Primary outcomes**			**WSI**					

			*Sensitivity*			87.9%					
			*Specificity*			95.7%					
			*Positive-predictive values*			97.1%					
			*Negative-predictive values*			82.7%.					
*Diagnostic concordance*			87% (163/186)^g^					

Ribback et al.^25^	Urology, gynecology, and dermatopathology	Tumor diagnosis and assessment of surgical margin	**Primary outcomes**			**WSI**					

			*Sensitivity*			92.6%					
			*Specificity*			99.0%					
			*Positive-predictive values*			98.3%					
			*Negative-predictive values*			97.7%					
*Diagnostic concordance*			98.35%					

Tawfik et al.^26^	Gynecological pathology	Assessing if negative for intraepithelial lesion or malignancy	*Sensitivity (95% CI)*								

			**Diagnosis**			**WSI**					
			Bacterial vaginosis			92%					
			Trichomona vaginalis			91%					
Fungi			95%					

Tawfik et al.^28^	Gynecological pathology	Diagnosing for neoplasms, cellular changes, and infectious agents according to 2001 Bethesda reporting system and terminology			**Weighted average for WSI (95% CI)**						
			**Diagnosis**		*Sensitivity*			*Specificity*			

			Atypical squamous cells of undetermined significance (ASCUS)		58.3%			85.1%			
			Low-grade squamous intraepithelial lesions (LSIL)		54.1%			93.9%			
			High-grade squamous intraepithelial lesions (HSIL)		51.8%			98.8%			
			Atypical glandular cells of undetermined significance (AGUS)		32.8%			99.1%			
			Atypical squamous cells, cannot exclude high-grade squamous intraepithelial lesion (ASC-H)		23.5%			99.5%			
Any condition^h^		82.1%			86.2%			

Tissier et al.^22^	Nephropathology	Classification of adrenocortical tumor by Weiss score^i^	**Primary outcomes**		**Reading 1**^j^			**Reading 2**			

			*Sensitivity (95% CI)*		86%			94%			
*Specificity (95% CI)*		100%			93%			

Zoroquiain et al.^23^	Ocular pathology	Identification of prognostic factors for retinoblastoma		**Morphological risk factors**			**Classic morphological features**				
			**Primary outcomes**	Optic nerve invasion	Invasion and spread		Growth pattern of retinoblastoma		Calcification		

			*Sensitivity*	100%	100%		100%		97.8%		
*Specificity*	100%	100%		100%		100%		

a Area under the curve (AUC) is the probability where the test with the target condition will have a higher value than the test without the target condition. It is represented with values from 0 to 1 and not in percentage^23^.

b Histopathological features are the main diagnostic findings that help to grade meningioma.

c Above consensus means over-interpretation of the test to a higher breast cancer stage.

d Below consensus is the opposite, under-interpretation to a lower stage.

e Pathologist interpretation is used to denote the comparison during the validation study between WSI and LM, where pathologists have used both technologies.

f Range of percentages in diagnostic concordance not reported.

g Range of diagnostic concordance consists in the ratio of the cases that agreed with the consensus diagnosis and the total number of cases.

h This is the average performance of WSI for all the above diagnostic categories but adjusted for the number of cases for each of the category.

i Weiss score is a reference method to distinguish between a benign and a malignant adrenocortical tumor (ACT).

j The study was designed in two stages of using WSI for the examination of the sample and the term ‘reading’ is used by the authors.

In a study of pancreatic pathology, Larghi et al. besides the overall good performance of WSI for sensitivity, specificity, and positive-predictive values, also reported a poor performance for negative-predictive values for both LM and WSI (51% and 52%, respectively).^24^ However, the authors do not explain the reasons for this poor performance.

One study of gynecological pathology, diagnosing several diseases according to the 2001 Bethesda Report, stated a poor sensitivity of WSI for each of the individual diseases (23.5%–58.3%, see Table 3 for more details).^28^ However, they report a higher average sensitivity (82.1%) that is adjusted to the number of cases for each diagnostic category. Similarly, in a study of surgical neuropathology, Ammendola et al. reported a poor performance of both LM and WSI based on AUC (from 0.50 to 0.72) for several diagnostic features of meningioma.^27^

Elmore et al., focusing on breast cancer, report a high predictive value, for both LM and WSI, in identifying benign without atypia (97.1% vs 95.7%) and invasive breast cancer (97.7% vs 97.2%).^29^ However, they report an average performance for Ductal Carcinoma in Situ (DCIS) (69.6% LM vs 57.1% WSI) and a poor performance for atypia (37.8% vs 27.8%).

#### Diagnostic concordance

Six studies out of 12 reported the diagnostic concordance of WSI with LM^20^^,^^21^^,^^24^^,^^25^^,^^30^^,^^31^ (Table 3). Four of these, reported a high diagnostic concordance for WSI in the range 86%–98.35%. Nielsen et al. conducting a study in dermatopathology, report an average concordance for both LM and WSI, 72.4% vs 69.6%, respectively.^30^ The authors briefly elaborate on the poor performance of WSI for premalignant changes, where the main problems with accuracy (and concordance) were observed. This might explain the average concordance as opposed to an otherwise very good performance for DTA indicators (see the subsection above and Table 3). Finally, a study of breast cancer reported a varying mean concordance for different stages of breast cancer.^20^ Similarly with the other breast cancer study,^29^ the poor concordance was observed for atypia (37%), the very good concordance in invasive breast cancer (94%).^20^

#### Degree of overdiagnosis

The degree of overdiagnosis was not explicitly reported in any of the 12 studies. There are ongoing and recent discussions whether overdiagnosis should be defined as a diagnostic error,^32^, thereby captured by the Bayesian reasoning (2x2 table). As Brodersen et al. remark, overdiagnosis is not a false-positive result classified as diagnostic error that with further investigation can be determined as such; it is an abnormality that meets the pathological criteria of a disease.^16^ In one of the selected studies, Elmore and colleagues elaborate on overinterpretation for several grades of breast cancer on both WSI and LM.^29^ The term overinterpretation was used to denote the incorrect classification of a lesion to a higher stage. The authors of this study, calculated that 3% of the cases were overinterpreted as invasive breast cancer with WSI, thereby overdiagnosed.

#### Additional outcomes

Six studies out of 12 reported on observer variability^22^^,^^24^^,^^26^^,^^27^^,^^29^^,^^30^ (Table 4). Of these, 4 studies tested intra or interobserver variability with Cohen’s kappa (к) statistics,^22^^,^^24^^,^^26^^,^^30^ and 2 in percentage.^27^^,^^29^ Two studies calculating intra- and interobserver variability based on к statistics, where the values for both LM and WSI were within к 0.67–0.97.^24^^,^^30^ The 2 other studies calculated к jointly for LM-WSI for different diagnostic features or categories, where interobserver variability was from к 0.21–0.83.^22^^,^^26^ Two studies reported the percentage of observer variability for LM and WSI, where intraobserver variability was from 73% to 100% for both.^27^^,^^29^ While, Ammendola et al. calculated also interobserver variability for senior pathologists (range 49%–97%) vs all observers (range 26%–93%) and all observers for LM (range 27%–83%) and WSI (31%–89%).^27^

**Table 4:** Additional outcomes for intra- and interobserver variability

Source		Secondary outcome						

Ammendola et al.^27^	Surgical neuropathology	*Intraobserver variability* between *LM & WSI*						

		**Histopathological features**	**Observer 1**	**Observer 2**	**Observer 3**	**Observer 4**		**Median**

		Atypical meningioma	91%	86%	74%	94%		89%
		Brain invasion	100%	91%	86%	97%		94%
		High mitotic index	80%	79%	77%	71%		78%
		Hypercellularity	94%	82%	97%	91%		93%
		Sheeting	97%	97%	77%	94%		96%
		Macronucleoli	94%	82%	100%	83%		89%
		Small cells	97%	94%	97%	91%		96%
		Spontaneous necrosis	97%	91%	94%	94%		94%

		*Interobserver variability between all observers (AO) and senior pathologists (SP)*^a^						

			**LM**			**WSI**		
		**Parameter**	**All observers**	**Senior pathologists**		**All observers**	**Senior pathologists**	

		Atypical meningioma	54%	63%		60%	74%	
		Atypical for major criteria	69%	86%		80%	86%	
		Atypical for minor criteria	46%	60%		63%	77%	
		Brain invasion	83%	97%		93%	97%	
		High mitotic index	80%	86%		69%	80%	
		Hypercellularity	74%	77%		86%	86%	
		Sheeting	57%	74%		66%	77%	
		Macronucleoli	37%	49%		40%	51%	
		Small cells	34%	49%		34%	49%	
		Spontaneous necrosis	26%	51%		31%	54%	
		*Interobserver variability for all observers*						

		**Parameter**		**LM**		**WSI**		

Brain invasion		83%		89%		
High mitotic index		80%		69%		
Hypercellularity		74%		86%		
Sheeting		57%		66%		
Macronucleoli		37%		40%		
Small cells		34%		34%		
Spontaneous necrosis		27%		31%		

Elmore et al. 2017^29^	Breast pathology	**Intervention**^b^			*Intraobserver variability*			

		LM VS LM			79%			
		WSI VS WSI			73%			
		LM VS WSI			77%			
WSI VS LM			76%			

Larghi et al.^24^	Pancreatic pathology			*Intraobserver variability*	*Interobserver variability*			
		**Parameters**^c^		**LM-WSI**	**LM**		**WSI**	

		Diagnostic classification		к^d^ = 0.87, 95% CI 0.81−0.93	84.5% [к 0.79; CI 0.71–0.88]		83.5% [к 0.78; CI 0.69–0.87]	
		Presence of core tissue		к = 0.68, 95% CI 0.59−0.77	79.3% [к 0.59; CI 0.45–0.72]		76.3% [к 0.53; CI 0.40–0.66]	
		Number of lesional cells		к = 0.67, 95% CI 0.56−0.77	74.3% [к 0.62; CI 0.52–0.71]		68.7% [к 0.53; CI 0.43–0.63]	
		Percentage of lesional cells		к = 0.77, 95% CI 0.71−0.83	50.2% [к 0.40; CI 0.30–0.50]		50.2% [к 0.38; CI 0.28–0.47]	
Mean			78.3% [к 0.67; CI 0.57–0.78]		77.8% [к 0.67; CI 0.57–0.77]	
Nielsen et al.^30^	Dermatopathology		*Intraobserver variability*				*Interobserver variability*	
		**Intervention (к statistics)**	**Pathologist 1**	**Pathologist 2**	**Pathologist 3**	**Pathologist 4**	**Reading 1**^e^	**Reading 2**

		LM	0.91	0.94	0.91	0.97	0.84	0.81
WSI	0.97	0.86	0.95	0.95	0.85	0.82

Tawfik et al.^26^	Gynecological pathology	*Interobserver variability (*к *statistics LM VS WSI)*						

		**Diagnosis**	**Reviewer 1**	**Reviewer 2**	**Reviewer 3**	**Reviewer 4**	**Reviewer 5**	**Weighted mean**

		Negative^f^ (95% CI)	0.74 (0.67–0.80)	0.49 (0.39–0.60)	0.63 (0.52–0.73)	0.79 (0.70–0.87)	0.61 (0.52–0.70)	0.68
		Atypical squamous cells of undetermined significance (ASCUS) (95% CI)	0.46 (0.39–0.52)	0.21 (0.10–0.32)	0.36 (0.25–0.46)	0.45 (0.36–0.44)	0.33 (0.24–0.43)	0.39
Low-grade squamous intraepithelial lesions (LSIL) (95% CI)	0.53 (0.47–0.59)	0.41 (0.31–0.52)	0.52 (0.42–0.63)	0.55 (0.46–0.64)	0.51 (0.42–0.60)	0.51
High-grade squamous intraepithelial lesions (HSIL) (95% CI)	0.58 (0.52–0.64)	0.36 (0.26–0.46)	0.42 (0.31–0.52)	0.58 (0.49–0.67)	0.54 (0.45–0.63)	0.52

Tissier et al.^22^	Nephropathology		*Intraobserver variability (Weiss score*^g^*criteria reading)*		*Interobserver variability (Weiss score criteria reading)*			
		**Diagnostic features**	**Reading 1**		**Reading 1**		**Reading 2**	

		Weiss≥3 vs 0–2	0.83		0.70 (0.67–0.74)		0.75 (0.72–0.79)	
		Necrosis	0.75		0.78 (0.74–0.81)		0.83 (0.79–0.86)	
		≤25% clear cells	0.42		0.71 (0.68–0.75)		0.80 (0.77–0.83)	
		Venous Invasion	0.58		0.54 (0.50–0.57)		0.54 (0.50–0.57)	
		Mitotic figures	0.42		0.54 (0.50–0.57)		0.65 (0.62–0.69)	
		Capsular Invasion	0.25		0.49 (0.45–0.52)		0.50 (0.47–0.54)	
		Diffuse architecture	0.33		0.41 (0.37–0.44)		0.50 (0.46–0.53)	
		Nuclear grade	0.25		0.39 (0.36–0.43)		0.45 (0.41–0.48)	
		Atypical mitotic figures	0.25		0.29 (0.26–0.33)		0.46 (0.43–0.50)	
		Sinusoidal invasion	0		0.40 (0.37–0.44)		0.30 (0.27–0.33)	
Weiss modified by Aubert et al ≥3 vs 0–2	0.50		0.67 (0.64–0.70)		0.75 (0.72–0.78)	

a Interobserver concordance was measured between all the observers (pathologists), but also between senior pathologists versus all the observers that participated in the validation study.

b Here all the possible combination of comparisons between LM and WSI were tried based on intraobserver agreement.

c Beside the diagnostic classification, in this study other diagnostic features were considered, therefore we use the term “parameters”.

d Kappa (к) statistics is used to assess observer agreement for intervention(s).

e At Nielsen et al., they use the term ‘review’ instead of ‘reading’. We have chosen the latter for a consistent terminology (as it is used e.g. in Tissier et al.).

f The case does not have the target condition.

g Weiss score is a reference method to distinguish between a benign and a malignant adrenocortical tumor (ACT).

## Discussion

The selected studies in this systematic review displayed a low risk of bias and applicability concerns as measured with the QUADAS-2.^18^^,^^19^ We found that WSI was not inferior to LM regarding diagnostic performance. In addition, in 4 studies reporting both LM and WSI, their performances were comparable.^24^^,^^27^^,^^29^^,^^30^ Moreover, 8 out of 12 studies state an overall very good performance of WSI regarding DTA and diagnostic concordance. However, the degree of overdiagnosis was not reported in any of the selected studies, which might have an impact on artificially increasing the performance of WSI like other newer imaging tests. In this regard, Heleno et al. assessing the accuracy of low-dose CT scans for lung cancer screening, found that overdiagnosis inflated sensitivity and positive-predictive values.^13^

The 12 studies included in the present review displayed a high heterogeneity and from the analysis of the data extracted, it seems that this has implications for the diagnostic performance of WSI in the validation studies of pathology. There are 3 main aspects, in addition to the risk of overdiagnosis, where heterogeneity played an important role regarding performance: study design, subspeciality, and sample preparation.

### Study design

The included studies design were quite diverse regarding the main CAP recommendations such as the number of samples, pathologists, washout period, order of examination with LM and WSI, and the comparison between them. Therefore, a reliable diagnostic performance is directly related to the quality of the validation study, as also remarked in another systematic review comparing WSI with LM.^33^ In line with Goacher et al., the quality of the evidence regarding WSI performance is hampered by the heterogeneity of the study design, despite the evidence that WSI was not inferior to LM.^34^ Thus, in our review 4 studies did not have a sufficient (60 cases) number of samples as recommended by CAP,^20^^,^^22^^,^^23^^,^^27^ which might have increased the uncertainty due to broader confidence intervals. Notwithstanding the low risk of bias and applicability, 6 studies did not report on the confidence intervals regarding the diagnostic performance of WSI or LM.^21^^,^^23^^,^^25^^,^^27^^,^^30^^,^^31^ This brings further questions about the sample size and whether it is representative of the population.

### Subspeciality

The included 12 studies represent different pathology subspecialties, and 2 even reporting on multiple subspecialties.^25^^,^^31^ Each subspecialty involves specific challenges regarding the number and type of diagnostic categories, as well as those cases requiring additional molecular tests for the final diagnosis.

For instance, Ammendola et al. reported AUC values (for both LM and WSI) evaluating atypical meningioma mostly in the range of 0.50–0.60.^27^ These values indicate a poor performance regarding test accuracy. Nonetheless, the authors concluded that the suboptimal performance regarding the grading of meningioma was due to the diagnostic challenges that this disease poses for pathologists. In this case, more experienced senior pathologists performed significantly better than younger ones. This finding has implications about the role of clinical reasoning in diagnostic accuracy, where the literature suggests expertise might be related with experience especially with pattern recognition of importance in visual diagnostics.^32^^,^^35^^,^^36^

Parallel to the increasing complexity of examinations, the subspecialty of gynecological pathology was challenged by a high diagnostic workload.^37^ In 2 studies of this subspecialty, the authors assessing the performance of WSI based on DTA indicators, evaluated 335^28^ and 1110^26^ slides. In one of the studies, the WSI showed high sensitivity for assessing intraepithelial lesions or malignancies.^28^ While, the other study displayed an inconsistent sensitivity for multiple diagnostic categories, but stated that their method of assessment was as sensitive as the standard reference method.^26^

Girolami et al. asserted that diagnostic performance is related to the time for making the diagnosis in cytology-based subspecialties.^37^ In this regard, Tawfik et al. reported an average scanning and reviewing time of 5.5 min with WSI for cytology-based gynecological pathology.^26^ In 3 other studies measuring the time for diagnosis with WSI, 2 stated that turnaround time (time of the arrival of the specimen until the communication of diagnosis) was comparable between LM and WSI,^25^^,^^31^ while Larghi et al. reported a comparable time for reviewing slides with LM and WSI, 84 and 108 s, respectively.^24^

### Sample preparation

Sample preparation techniques pose specific challenges for slide digitization that might affect the performance of WSI, both regarding accuracy and time. One such example are cytology preparations – where smear thickness, overlapping cells, and obscuring backgrounds require multiplane (z-stacking) focusing for digital slides.^28^ From the selected articles, 3 of them were based on cytology preparations,^21^^,^^26^^,^^28^ 1 involved both cell-blocks (cytology) and histology samples,^24^ while 2 of them used frozen sections.^25^ Despite the difficulties of sample preparation, all these studies reported a comparable performance of WSI with LM.

This important aspect of using WSI with z-stacking for routine work with cytology preparations was also emphasized in a systematic review of digital pathology for cytopathology.^37^ However, one study of surgical neuropathology based on histology preparations used 7 z-stack planes and a technique for optimizing the digital slide.^27^ Notwithstanding the fact that histology is less challenging for digitization, the performance of pathologists was not more accurate than with LM. However, even with single or multiple z-stacking, cytopathology and frozen sections are still difficult to digitize with a high quality of image as it can be achieved with histopathology slides.

### Overdiagnosis

Adding to the challenges relating to diagnostic performance and the role of heterogeneity, overdiagnosis poses other difficulties. Although its degree was not reported explicitly, it was briefly addressed in the 2 breast cancer studies.^20^^,^^29^ Brunyé et al. mention the notion of overdiagnosis, by elaborating on its unnecessary and costly treatment and intervention procedures, for instance, when a biopsy is interpreted as ductal carcinoma in situ (DCIS) when in fact is atypia.^20^ Conversely, Elmore et al. calculated the number of cases incorrectly classified to a higher stage (per hundred cases), showing that 3% with WSI and 2% with LM (as the reference standard) of cases were overinterpreted as invasive breast cancer.^29^ However, this was a validation study scenario, where clinical outcomes were not calculated, but only the performance of the pathologists involved in this study. In this regard, future studies should evaluate the DTA of WSI by including *patient-relevant outcomes,* and thereby overdiagnosis in a randomized design to encompass the full spectrum of cases.^29^

While there are 5 cancers documented with high risk of overdiagnosis, the reasons for each of them are different such as screening (i.e., breast cancer, prostate cancer, and melanoma), incidental findings (renal cancer), or both incidental findings and excessive investigation (thyroid cancer).^38^ However, there are other cases such as lung cancer, where overdiagnosis is possible if screening for lung cancer is implemented.^39^ In this review, we focused on pathological diagnostics by comparing WSI to LM and not on the above factors for overdiagnosis. In this regard, the Cochrane Collaboration has launched a new research field regarding the use of evidence to tackle overdiagnosis and its consequences.^40^

### Shortcomings of the systematic review

The heterogeneity of the included studies hindered the possibility of conducting a meta-analysis, thereby limiting the comparative power of our study. While this could have provided a quantitative summary of the diagnostic performance of WSI in comparison to LM, the descriptive analysis in this review provided a qualitative account for it. The combination of at least 2 primary outcomes as the main criteria for selection, limited the number of the included studies. However, this was a methodological choice to include several accuracy measurements (i.e., DTA indicators, diagnostic concordance, and observer variability) for assessing the diagnostic performance of WSI. Ultimately, the question whether WSI should be implemented for routine work in pathology depends on how WSI addresses the logistical and organizational challenges that pathology departments face and the opportunities they afford. While, the opportunities of using digital pathology solutions are increasingly related with the use of AI for image analysi,s^6^^,^^7^ in this review, we do not address this aspect.

### Implications for practice

With a continuing shortage of pathologists and the multiple challenges that these departments face, digital pathology presents some opportunities to address them. Remote work and consultations^5^ through WSI are often presented as a good solution to address the lack of pathologists and a growing workload. Following this, the possibility to train residents and pathologists with this digital solution adds to the capacity building in order to tackle these challenges.^2^ Finally, the prospect of using AI algorithms for quantitive measuring, counting, and computer-assisted diagnosis might contribute in better diagnostic accuracy and saving time for pathologists.^4^^,^^7^

## Conclusion

We found that WSI was not inferior to LM regarding DTA and diagnostic concordance. However, the degree of overdiagnosis was not systematically reported and is thereby unknown. The diverse subspecialties and their laboratory tasks pose important questions whether it is possible to compare LM and WSI across all these subspecialties, or that perhaps LM has advantages in some and WSI in others. When considering the implementation of digital pathology, departments should also take into account the advantages for remote diagnosis and consultations, cancer research, digital multidisciplinary case conferences, supervision of residents, and storage of digital slides. However, the designers of the validation studies and the participating pathologists should be careful in those areas where the risk of overdiagnosis exists.

## Funding support

This research did not receive any specific grant from funding agencies in the public, commercial, or not-for-profit sectors.

## Authors’ contributions

OK and JBB conceptualized the systematic review. The other authors helped to refine conceptualization before submitting the protocol. Database search, screening, data extraction, risk of bias, data analysis and synthesis, were conducted independently by CVR and OK. JBB acted as an arbiter in cases of disagreement. ESR helped with the terminology in the study and his expertise as a senior pathologist throughout different steps. TR helped with the writing and reviewing the manuscript of the review. OK and CVR wrote the first draft and all the other authors helped during the writing, editing, and reviewing process.

## Conflicts of interests

The authors declare no conflicts of interests.

## Appendix A Supplementary data

Supplementary material Image 72
