# Supplementary material for: Lost in digitization – A systematic review about the diagnostic test accuracy of digital pathology solutions
Source: J Pathol Inform. 2022 Sep 6;13:100136. doi: 10.1016/j.jpi.2022.100136 (PMC9577136; doi:10.1016/j.jpi.2022.100136)
Supplement: Supplementary file 1 — Supplementary material [file mmc1.pdf]

Supplementary Table 1. General Information and PICO[a] for the selected studies.

General information		(PICO) Population				Reference standard	Intervention			Outcomes[b]	
**Study**	**Country**	**Subspecialty**	**Diagnostic category**	**Specimen**	**Stain**	**Light microscope (LM)**	**WSI**	**scanner**	**Features**	**Primary**	**Additional**
Ammendola et al.[27]	Italy	Surgical neuropathology	Meningioma	FFPE[c] tissue from resected specimens	HE[d]	Nikon Eclipse 80i	Hamamatsu photonics	NanoZoomer S260	Magnification X40, 7 z-stacking planes	Area Under the curve (AUC)	Intra- and interobserver variability
Brunyé et al.[20]	US	Breast pathology	Neoplasms of the breast	FFPE biopsy	HE	Consensus diagnosis with LM	Microsoft silver light platform	iScan Coreo Au scanner	Magnification x40, Scanning time <12 min for 5cm^2 tissue section, Capacity 2 slides	Diagnostic concordance	NA
Cima et al.[31]	Italy	Multiple	Neoplasms and organ quality (after transplantation)	Archived frozen sections	HE	Reference diagnosis with LM (not specified)	Visia Imaging	Navigo	Magnification x20	Sensitivity, specificity, predictive values (positive and negative), diagnostic concordance	NA
Elmore et al.[29]	US	Breast pathology	Neoplasms of the breast	FFPE biopsy	HE	Consensus diagnosis made with both LM and WSI	Microsoft silver light platform	iScan Coreo Au scanner	Magnification x40	Predictive values	Intraobserver variability
Larghi et al.[24]	Italy	Pancreatic pathology	Pancreatic solid lesions	FNA/FNB[e] prepared as either cell block or direct FFPE	HE	Reference diagnosis with LM (not specified)	Leica biosystems	Aperio ScanScope XT	Magnification x20	Sensitivity, specificity, predictive values (positive and negative), diagnostic concordance	Intra- and interobserver variability
Nielsen et al.[30]	Denmark	Dermatopathology	Neoplasms of the skin	FFPE biopsy (excision biopsy, shave biopsy, and curettage)	HE	Type of LM not specified	Carl Zeiss MicroImaging	Mirax Scan	Magnification x20	Sensitivity, specificity, predictive values (positive and negative), diagnostic concordance	Intra- and interobserver variability
Perez et al.[21]	US	Not specified	Neoplasms	Touch imprints from needle core biopsies (NCB)	Diff-Quick stain[f]	Comparison to final NCB diagnosis with LM	Olympus	CX41 microscope and digital camera with	NA	Sensitivity, specificity, predictive values (positive and negative), diagnostic concordance	NA

								NetCam software			
Ribback et al.[25]	Germany	Urology, gynecological and dermatopathology	Neoplasms	FFPE tissues slides	HE	Type of LM not specified	Carl Zeiss MicroImaging	Mirax Scan	Magnification x20, Time for scanning 0.5-0.7 MB/sec	Sensitivity, specificity, predictive values (positive and negative), diagnostic concordance	NA
Tawfik et al.[26]	US	Gynecological pathology	Negative for intraepithelial lesion or malignancy	Pap smear cell block	HE	Consensus cytology diagnosis, Type of microscope not reported	Aperio Technologies	Aperio ScanScope XT	Magnification x 40	Sensitivity	NA
Tawfik et al.[28]	US	Gynecological pathology	Neoplasms, cellular changes, and infectious agents	Pap smear cell block	HE	Consensus cytology diagnosis, Type of microscope not reported	Aperio Technologies	Aperio ScanScope XT	Magnification x 40	Sensitivity, Specificity	Interobserver variability
Tissier et al.[22]	France	Nephropathology	Neoplasm	FFPE tissue from resected specimens	HE	Type of LM not specified	Aperio Technologies	Aperio ScanScope XT	Magnification X40	Sensitivity, Specificity	Intra- and interobserver variability
Zoroquiain et al.[23]	Spain	Ocular pathology	Retinoblastoma	FFPE	HE	Type of LM not specified	Ventana	iScan Coreo Au scanner	Magnification x40, Capacity 160 slides	Sensitivity, Specificity	NA

a. PICO – Population, Intervention, Comparison (or reference standard), and outcomes.
b. Outcomes are reported on Table 1 (primary) and Table 2 (additional).
c. FFPE – Formalin-fixed and paraffin embedded.
d. HE – hematoxylin and eosin stains.
e. FNA – fine needle aspiration, FNB – fine needle biopsy.
f. Diff-Quick – Romanov and Giemsa commercial stains.

Supplementary Table 2. CAP recommendations and time for diagnosis.

**General information**	**CAP recommendations[a]**					**Turnaround time[b]**
**Study**	**Nr of cases**	**Nr of Pathologists**	**Washout period**	**Order of evaluation**	**Technical discrepancies**	**Time for diagnosis**
Ammendola et al.[27]	35	4	3-6 weeks	LM and then WSI	NA	NA
Brunyé et al.[20]	24	40	NA	LM by consensus panel followed by WSI	NA	Time reported only for WSI and it was 192.6 seconds
Cima et al.[31]	125	1	3 weeks	LM and then WSI	11 out of 124 slides needed a second scan, while 4 out of 125 slides were excluded because of failed digitization.	Time reported only for WSI regarding Cancer staging: mean 12 minutes (range 4-30), and Transplantation: mean 18 minutes (range 3-40)
Elmore et al.[29]	240	208	9 months	Randomized for both LM and WSI	NA	NA
Larghi et al.[24]	60	5	3 months	LM and then WSI	NA	LM 84 seconds, WSI 108 seconds
Nielsen et al.[30]	96	4	3 weeks	WSI and then LM	NA	NA
Perez et al.[21]	186	1	NA	WSI and then LM	There were 6 cases with loss of diagnostic material	NA
Ribback et al.[25]	293	NA	NA	WSI and then LM	NA	Time reported only for WSI 35.6 +/- 1.65 minutes per case
Tawfik et al.[26]	335	6	NA	LM and then WSI	NA	NA
Tawfik et al.[28]	1110	5	NA	LM and then WSI	NA	Time reported only for WSI with 3 minutes (range 2-8) for review, Case scanning time averaged 2.5 minutes
Tissier et al.[22]	50	12	NA	NA	NA	NA
Zoroquiain et al.[23]	47	NA	10 days	LM and then WSI	NA	NA

a. These are the main recommendations from the College of American Pathologists (CAP) that we extracted in our selected studies. However there are other recommendations that they put forth in [1].
b. Turnaround time encompasses the whole process of receiving the sample, preparing the slides (glass or digital), and signing out the diagnostic report. Here is used to include scanning time and diagnostic work.
